# Facilitation Effects of *Haloxylon salicornicum* Shrubs on Associated Understory Annuals, and a Modified “Stress-Gradient” Hypothesis for Droughty Times

**DOI:** 10.3390/plants9121726

**Published:** 2020-12-07

**Authors:** Nasr H. Gomaa, Ahmad K. Hegazy, Arafat Abdel Hamed Abdel Latef

**Affiliations:** 1 Department of Botany and Microbiology, Faculty of Science, Beni-Suef University, Beni-Suef 62521, Egypt; 2 Biology Department, College of Science, Jouf University, P.O. Box 2014, Sakaka 72341, Saudi Arabia; 3 Department of Botany and Microbiology, Faculty of Science, Cairo University, Giza 12613, Egypt; hegazy@sci.cu.edu.eg; 4 Department of Biology, Turabah University College, Turabah Branch, Taif University, P.O. Box 11099, Taif 21944, Saudi Arabia; a.moawd@tu.edu.sa; 5 Botany and Microbiology Department, Faculty of Science, South Valley University, Qena 83523, Egypt

**Keywords:** desert, facilitation effects, soil characteristics, stress-gradient hypothesis, species richness, rainfall

## Abstract

Perennial shrub-annual plant interactions play key roles in desert regions influencing the structure and dynamics of plant communities there. In the present study, carried out in northwestern Saudi Arabia, we examined the effect of *Haloxylon salicornicum* shrubs on their associated understory annual species across four consecutive growing seasons, along with a record of the seasonal rainfall patterns. We measured density and species richness of all the annual species in permanent quadrats located beneath individual shrubs, as well as in the spaces between shrubs. During wet growing season *H. salicornicum* shrubs significantly enhanced the density and species richness of sub-canopy species, whereas in the relatively dry seasons they exerted negative effects on the associated species. In all growing seasons, the presence of shrubs was associated with enhanced soil properties, including increased organic carbon content, silt + clay, and levels of nutrients (N, P and K). Shrubs improved soil moisture content beneath their canopies in the wet growing season, while in the dry seasons they had negative effects on water availability. Differences in effects of *H. salicornicum* on understory plants between growing seasons seem due to the temporal changes in the impact of shrubs on water availability. Our results suggest the facilitative effects of shrubs on sub-canopy annuals in arid ecosystems may switch to negative effects with increasing drought stress. We discuss the study in light of recent refinements of the well-known “stress-gradient hypothesis”.

## 1. Introduction

Vegetation in arid land is often arranged in a two-phase spatial mosaic, composed of shrub patches interspersed in a bare ground matrix [1]. Biological interactions including facilitation and competition often occur between perennial shrubs and associated plant species living beneath their canopies [2,3]. These interactions are key ecological processes which affects composition, structure and diversity of plant communities in all terrestrial ecosystems [4,5]. 

Facilitation has been shown to be more frequent in “stressful” environments such as deserts, where it is invoked as a force in driving the structure of natural communities and regional vegetation dynamics [6]. Facilitation is regarded as a net positive effect of one plant species on another [7]. In facilitative interactions, shrubs serve as a kind of “nurse plant”, acting to decrease the environmental severity experienced by its understory plants [8], and thereby increasing their recruitment, growth, survival and reproductive success [9]. Moreover, in harsh environments dominant shrubs also enhance species richness of plant communities [10] through reducing the environmental stress beneath their canopies and thus promote the presence of species in habitats marginal to their physiological tolerances [11].

In deserts, shrubs facilitate their under-canopy plants through various mechanistic pathways. These include increasing nutrient availability, reducing air and soil temperature, decreasing soil water evaporation and transpiration of understory individuals, increasing water availability through hydraulic lift or reduction of evaporation, protection from photo-inhibition due to interception of direct solar radiation, protection against herbivory and trapping wind-dispersed seeds from nearby open areas [12]. Nutrients beneath shrubs are enhanced due to litter fall, soil particles accumulation, higher mineralization rates, and larger microorganism populations compared to interspaces [13].

Many studies of facilitation interactions over the past two decades have been stimulated by the “stress-gradient hypothesis”, which argues that the frequency and importance of facilitation increase with increasing environmental stress [14]. Predictions of the stress-gradient hypothesis have been widely supported by many empirical studies [15,16], though some recent studies showed that facilitation does not increase monotonically with increasing severity of physical conditions [17,18]. Thus, the stress-gradient hypothesis has been refined to show that facilitative effects decrease or cease or may even be reversed under extreme stress levels [19,20,21]. The differences between the predictions of the original and the recent refinements may originate from variation among studies regarding the type of stress gradient, the spatial and temporal scale of the study, and the particular performance measures considered. Despite the relatively large number of studies testing the stress-gradient hypothesis, some uncertainty remains, in particular in harsh ecosystems, about whether the intensity of facilitation increases with the environmental stress [19].

Annual plants constitute the main component of vegetation making up species richness in arid regions, and they possess important ecological functions [22]. Shrubs can affect the micro-distribution of annuals in deserts by creating different microhabitats, through modifying the spatial distribution of resources and conditions. They may also provide understory annuals with refuge from high solar radiation and soil water stress, leading to greater richness and density of annual species under shrub canopies relative to open areas between them [12,23].

Rainfall is the major limiting factor controlling seed germination, growth and productivity of annual plants in xeric ecosystems; this is because of its scarcity, unpredictability and variability in space and time [24]. Based on the assumptions of the stress-gradient hypothesis and its recent refinements, it can be hypothesized that the intensity of facilitation will vary with temporal variations in annual precipitation. Understanding the relationships between plant interactions and precipitation fluctuation in desert regions will help to predict the responses of vegetation to future rainfall changes associated with the predicted global climate change [21].

Many studies have assessed plant interactions in different locations along gradients in spatial aridity (e.g., [25,26]) but few have investigated these interactions over a multi-year temporal drought gradient (but see [20,21]). Recent research has highlighted the importance of long-term field studies of shrub-understory interactions in arid habitats, because short-term and long-term effects of shrubs on their sub-canopy plants may be different [27].

In the present study, we tested the following hypotheses: (1) shrubs promote abundance and species richness of annual plants under their canopies; and (2) the beneficial facilitative effects of shrubs on under-canopy annuals will diminish or even become negative under severely low rainfall. To test these, we conducted a study in an arid region in northwestern Saudi Arabia over four consecutive growing seasons differing in the amount of rainfall. This will assess effects of the dominant regional shrub, *Haloxylon salicornicum* (Moq.) Bunge ex Boiss. on the abundance, species richness and community structure of understory annual plants.

## 2. Results

**Figure 1:**
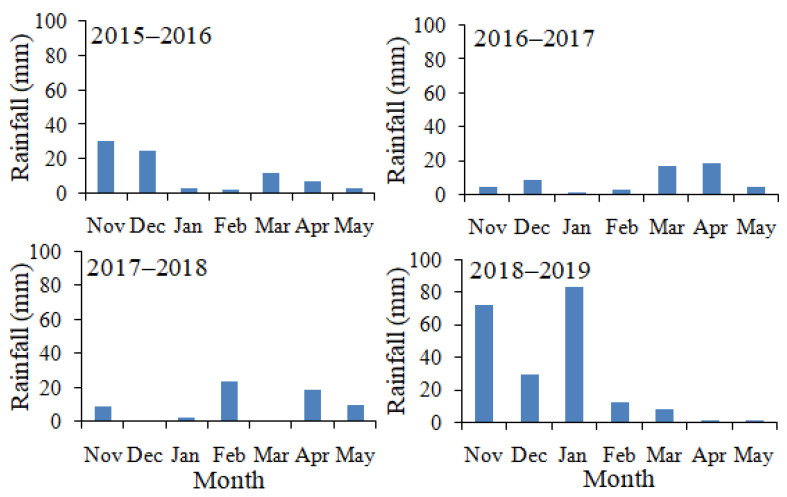
Monthly rainfall at the study area during four growing seasons.

Annual rainfall in the study area typically occurs mainly in November and extends through May (Figure 1). The extent of rainfall differed between the four growing seasons of the present study. Total rainfall was 82.1 mm in the 2015–2016 season, 56.9 mm in the 2016–2017 season, 64.3 mm in the 2017–2018 season and 207.3 mm in the 2018–2019 season.

Results of the general linear model (GLM) (Table 1) showed that the total density of plants was significantly affected by shrub and growing season. Likewise, the species richness was significantly influenced by the presence of shrub and growing season. The shrub × growing season interaction was significant for both total plant density and species richness.

**Figure 2:**
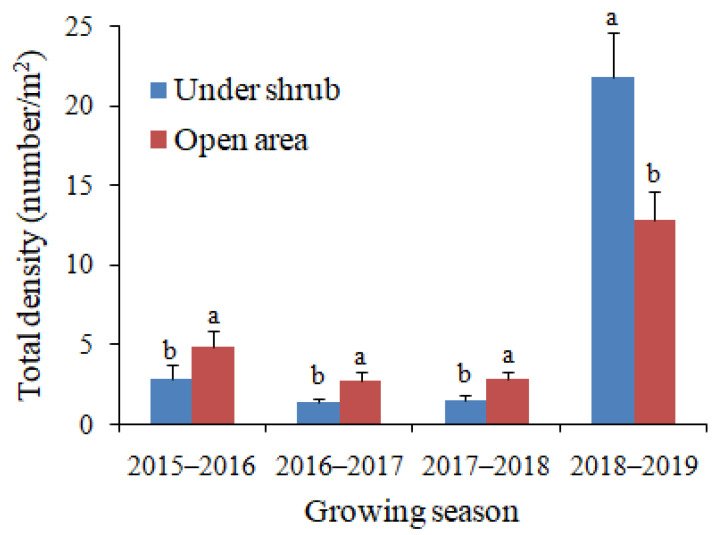
Effects of *Haloxylon salicornicum* shrubs on the total density of annual plants in four growing seasons. Values are means ± SD. Different letters indicate significant difference at *p* < 0.05 between under shrub and open area in each growing season.

**Figure 3:**
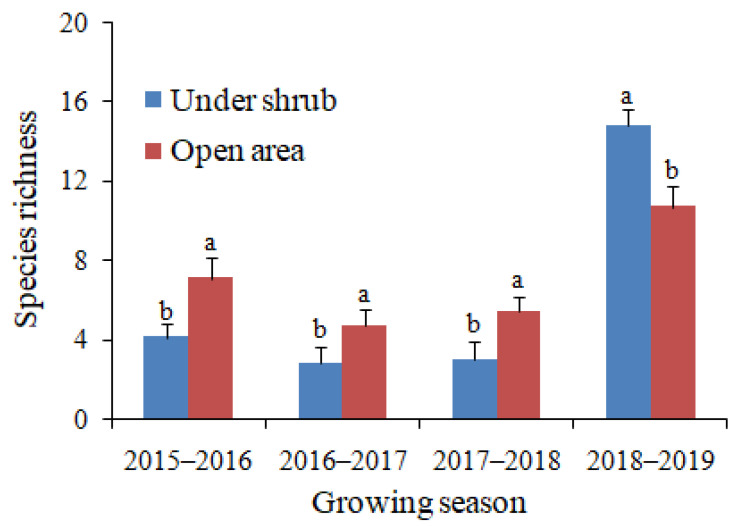
Effects of *Haloxylon salicornicum* shrubs on the species richness of annual plants in four growing seasons. Values are means ± SD. Different letters indicate significant difference at *p* < 0.05 between under shrub and open area in each growing season.

Both total plant density and species richness were significantly greater in open areas than under shrubs in the first three dry growing seasons of the study, 2015–2016, 2016–2017 and 2017–2018 (Figure 2 and Figure 3). By contrast, both parameters were significantly greater under shrubs than in open microhabitat in the fourth growing season, 2018–2019 (Figure 2 and Figure 3). In both microhabitats, the total density and species richness of annual plants were much greater in 2018–2019 than in the other growing seasons (Figure 2 and Figure 3). The total plant density ranged from low mean values of 1.4 and 2.7 individuals/m^2^ for under shrub and open area microhabitats, respectively in 2016–2017 to high values of 21.8 and 12.8 individuals/m^2^ for under shrub and open area, respectively in 2018–2019 (Figure 2). Likewise, the greatest values of species richness (14.8 and 10.7 for under shrub and open area, respectively) were observed in the growing season with the greatest amount of rainfall (2018–2019), while the lowest values (2.8 and 4.7 for under shrub and open area, respectively) were associated with the growing season with lowest rainfall (2016–2017) (Figure 3). Values of the mean relative interaction index (RII) were negative and significant for both total plant density and species richness in the first three growing seasons of our study, while in 2018–2019 the RII values were significantly positive (Table 2).

A total of 38 annual species were recorded over the four growing seasons of study (Table 3), 21 species in 2015–2016, 17 in 2016–2017, 16 in 2017–2018, and 38 in 2018–2019. Some 13 species were observed in all four growing seasons, whereas 11 were recorded in only one season, 12 in two seasons and 2 species in three seasons. The maximum number of recorded species was observed in the wettest growing season of the study (2018–2019). As indicated by RII values (Table 3), *H. salicornicum* shrubs had significant negative effects on 11 species (*Agriophyllum minus*, *Anthemis haussknechtii*, *Astragalus annularis*, *Bassia muricata*, *Ifloga spicata*, *Malva parviflora*, *Plantago boissieri*, *Plantago ciliata*, *Plantago ovata*, *Rumex vesicarius*, and *Schismus barbatus*) in 2015–2016; four species (*Agriophyllum minus*, *Anthemis haussknechtii*, *Plantago boissieri* and *Schismus barbatus*) in 2016–2017; and six species (*Anthemis haussknechtii*, *Bassia muricata*, *Erodium laciniatum*, *Plantago boissieri*, *Plantago ovata*, and *Schismus barbatus*) in 2017–2018. By contrast, 16 species were positively facilitated by the presence of *H. salicornicum* shrubs in 2018–2019 (Table 3).

Species-specific responses of annual plants to the perennial shrubs varied from growing season to season. For example some species, such as *Anthemis haussknechtii*, *Plantago boissieri*, and *Schismus barbatus* were significantly facilitated by *H. salicornicum* shrubs in the final growing season of the study, but showed an opposite response in the other growing seasons. Two species, *Bassia muricata* and *Plantago ovata* were facilitated by *H. salicornicum* shrubs in 2018–2019, but negatively affected by shrubs in 2015–2016 and 2017–2018, and not affected in 2016–2017. In response to the presence of *H. salicornicum* shrubs, the densities of *Ifloga spicata* and *Malva parviflora* were reduced in 2015–2016, increased in 2018–2019 and not affected in the other two growing seasons (Table 3).

**Figure 4:**
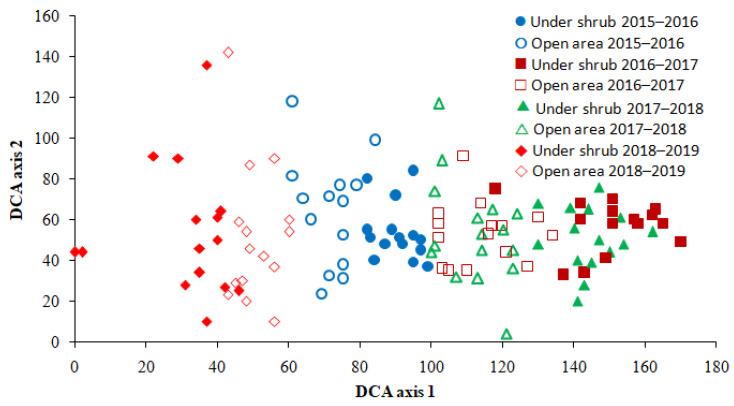
Detrended Correspondence Analysis (DCA) ordination of observations made in under shrub and open area microhabitats over four growing seasons (each plotted point represents a sampled stand in each growing season). Eigenvalue of DCA axis 1 = 0.614, eigenvalue of DCA axis 2 = 0.341.

The Detrended Correspondence Analysis (DCA) ordination showed the separation of six groups according to the microhabitat and growing season (Figure 4). These groups were arranged mainly along axis 1 from left to right in the order: under shrub 2018–2019, open area 2018–2019, open area 2015–2016, under shrub 2015–2016, open area 2016–2017 and 2017–2018, and under shrub 2016–2017 and 2017–2018. Observations representing the shrub microhabitat were separated from those of open area. Furthermore, observations made in the same microhabitat in different growing seasons were separated from each other except for the two growing seasons, 2016–2017 and 2017–2018 (Figure 4). The distribution of observations in the ordination plot suggests that the structure of annual plant community is affected by *H. salicornicum* shrubs, and varied among growing seasons.

The GLM analysis showed that the scores of DCA axis 1 were significantly affected by shrub and growing season (Table 4). The effect of shrub × growing season interaction was also significant for DCA axis 1 scores. The scores of DCA axis 2 were not influenced by shrub or growing season (Table 4). These results showed that the separation of observations along DCA axis 1 and hence the community structure was significantly influenced by microhabitat and growing season.

As indicated by results of the GLM analysis (Table 5), all measured soil properties except pH were significantly affected by the presence of shrub. Soil moisture content varied significantly in response to growing season, whereas other soil characters did not change between growing seasons. The shrub × growing season interactions were not significant for any soil parameters except soil moisture content. The properties of soils under shrub and in open areas across the four growing seasons are shown in Table 6. Organic carbon content, electrical conductivity, silt + clay, and nutrients (N, P and K) were significantly greater under shrubs than in open area in all four growing seasons. In contrast, sand content was lower under shrub canopy than in open microhabitat. Soil moisture content was significantly lower beneath shrubs than in the interspaces between them in the first three growing seasons of the study, while in the fourth season an opposite trend occurred (Table 6). Soil moisture generally increased with growing-season rainfall ranging from 1.18 and 1.35% in the driest growing season (2016–2017) to maximum values of 4.77 and 4.22% (for under shrub and open area, respectively) in the wettest growing season (2018–2019) (Table 6).

## 3. Discussion

In the present study, *H. salicornicum* shrubs enhanced the species richness and density of their understory annual plants in the wet growing season, 2018–2019. On the other hand, these shrubs had significant negative effects on their associated understory plants in the relatively dry growing seasons (2015–2016, 2016–2017 and 2017–2018).

Our results do not support the stress-gradient hypothesis, which suggests a monotonic increase in facilitation with environmental stress [15,16], but agree with its recent refinements predicting that the positive effects of shrubs may be shifted to neutral or negative at the extreme end of the aridity gradient [19,20,21]. The present results support our hypotheses that shrubs enhance the density and species richness of annual plants beneath their canopies, and that the facilitative effects of shrubs on annual plants change to negative under severely low rainfall.

The facilitative effects of shrubs in the wet growing season could be related to the greater soil moisture content beneath shrubs compared the open areas, whereas their negative effects in the dry growing seasons may be attributed to the lower soil moisture content below the perennial shrubs than in the interspaces around them. During small rain events, shrubs might decrease water availability under their canopies by intercepting rainwater, making the soil below them dryer than in interspaces [28,29,30]. Furthermore, the water intercepted by shrub crowns during small rain events is lost in wetting the canopy surface and evaporation into the atmosphere and is less likely to pass through the plant canopy [31]. In contrast, during moderate to heavy rain, shrubs direct water intercepted by their canopies to the understory through stemflow [29,32]. Moreover, the low evaporation rate below shrubs [21] may lead to greater moisture retention of the soil under shrubs than in soil from open areas. Thus, in growing seasons with moderate or heavy rains, water availability is greater beneath shrubs than in open area.

The departure of our results from the predictions of the stress-gradient hypothesis could be related to the assumption that the effects of shrubs on soil moisture change with variations in growing-season rainfall. The positive effects of shrubs on water availability of sub-canopy soil in the wet growing season shifted to negative influences in the relatively dry seasons. Zhang et al. [21] pointed out that shrub-herbaceous species interactions shifted from positive to neutral with increasing drought stress in the Badain Jaran desert, in China. To interpret this pattern, they suggested that the facilitative effects of shrub on soil moisture diminish with decreasing rainfall. In addition, O’Brien et al. [20] showed that the facilitative effects of *Retama sphaerocarpa* shrubs on species richness and plant productivity of the associated herbaceous community prevailed in growing seasons with more than 120 mm rainfall, and switched to negative effects under severe rainfall deficit when precipitation ranged between 70 and 120 mm. Some studies (e.g., [33,34]) have related the change from facilitation to negative interactions in water-limited ecosystems to competition for water between shrubs and their understory plants. This is unlikely to happen in our study system because the absorbing roots of *H. salicornicum* are deep, often extending beyond 5 m and reaching to 8–10 m deep [35], while those of annual plants are generally shallow.

The present results indicated that *H. salicornicum* shrubs significantly affected important soil properties. Compared to open areas, soils under canopies had greater values of silt + clay content, organic carbon content, electrical conductivity and concentrations of N, P and K. These results agree with those of Rathore et al. [36] in their study on the impact of *H. salicornicum* and *Calligonum polygonoides* shrubs on soil properties in arid Western Rajasthan, India. The accumulation of wind-blown, fine-soil particles under shrubs leads to an increase in silt + clay content [37]. The increase in organic carbon content and nutrient concentrations under shrubs may be due to the fact that shrubs decrease soil erosion and trap wind-blown, nutrient-rich materials [37]. The accumulation of litter on the ground under shrubs might also enhance nutrients availability below shrubs [38]. The increase in soil electrical conductivity beneath canopies may reflect the accumulation of salts in the litter [39]. The facilitative effects of *H. salicornicum* shrubs on soil nutrients and organic carbon suggest that using this species in vegetation restoration of degraded desert ecosystems could improve soil fertility, thereby enhancing species diversity. Rathore et al. [36] recommended the application of *H. salicornicum* to restore desertified arid lands in India. In addition to the greater water availability, the relatively higher soil fertility under *H. salicornicum* might contribute to the facilitative effect of shrubs on annual plants in wet growing seasons. The role of the higher levels of soil organic carbon and nutrients underneath shrubs in facilitating under-canopy plants has been reported in several studies in arid and semi-arid environments (e.g., [40,41,42]). In relatively dry growing seasons, the greater concentration of soil nutrients beneath *H. salicornicum* shrubs did not enhance the species richness and density of understory annual plants because water is generally a more limiting factor than nutrients for plants growing under arid conditions [43]. Tielbörger and Kadmon [28] stated that in severely low rainfall years, the beneficial effects of improved nutrients under shrubs are overshadowed by water limitation.

Although we did not estimate the density of individuals in the soil seed bank, the facilitative effect of *H. salicornicum* shrubs in the wet growing season may be also related to the greater size of soil seed bank under shrubs. In desert environments, shrubs have been shown to have increased seed bank size under their canopies through various mechanisms, including trapping seeds by shielding wind-dispersed seeds, and facilitating the accumulation of animal-dispersed seeds by acting as perch site for seed-carrying birds or as rodent cache [36,44]. The greater seed bank below shrubs had no role in enhancing the density and species richness of understory annual plants in the dry growing seasons because the amount of water available under shrubs is apparently too low to facilitate seed germination [45].

With the exception of soil moisture, the effects of *H. salicornicum* shrubs on observed soil characters did not change with variation in growing-season rainfall. This suggests these soil properties do not directly modulate the variation in shrub-annual plants interactions along temporal aridity gradients. Still, in wet years, the greater soil fertility, in addition to higher water availability, under shrubs may play a role in facilitating understory annual plants.

The responses of individual species to *H. salicornicum* shrubs were not consistent over different growing seasons. The species which were facilitated by shrubs in the wet 2018–2019 season were either negatively affected or not measurably affected by shrub presence in the other relatively dry growing seasons. Our results agree with those of Tielbörger and Kadmon [46] who indicated that the responses of annuals plants, in a sandy desert ecosystem, to shrub-opening gradients varied significantly among years of variable rainfall. Our findings are not consistent with those of other studies which have suggested that desert annual plants growing in shrub communities could be categorized into distinct groups based on their response to shrubs versus open areas (e.g., [47,48]). Tielbörger and Kadmon [46] showed that this classification was questionable since these studies observed the same community for only one year.

The results of DCA ordination and GLM suggest that the presence of *H. salicornicum* shrubs influences the structure and dynamics of the annual plant community, and that the community-level responses to shrubs were growing season-dependent. Significant effect of shrubs on the structure of understory annual plants have been reported in other studies in arid ecosystems [41,49]. The variation in the influence of shrubs in structuring annual plant communities between growing seasons appears related to the fact that shrubs may enhance the establishment of under-canopy annual species during one growing season and inhibit the establishment of the same species in another growing season [46]. The change in the effects of shrubs on the structure of understory plant community between growing seasons is at odds with the results of Shmida and Whittaker [47] who showed that shrubs produce a relatively stable pattern of microhabitat differentiation. The variation in both the species- and community-level responses to *H. salicornicum* shrubs between growing seasons appears to us to be related to temporal changes in the impact of shrubs on water availability. *H. salicornicum* shrubs had negative effects on under-canopy wetting in dry growing seasons, but enhanced understory soil moisture in the wet season.

Our results may help to explain the pattern of shrub-annual interactions and therefore, predict the vegetation change in response to the increasing aridity that is expected in several regions of the world in the coming decades [50,51,52]. The results suggest that the negative effects of shrubs on the abundance and species richness of their understory plants will predominate with the predicted increase in drought stress associated with climate change thereby putting biodiversity at risk.

## 4. Materials and Methods

### 4.1. Study Area

**Figure 5:**
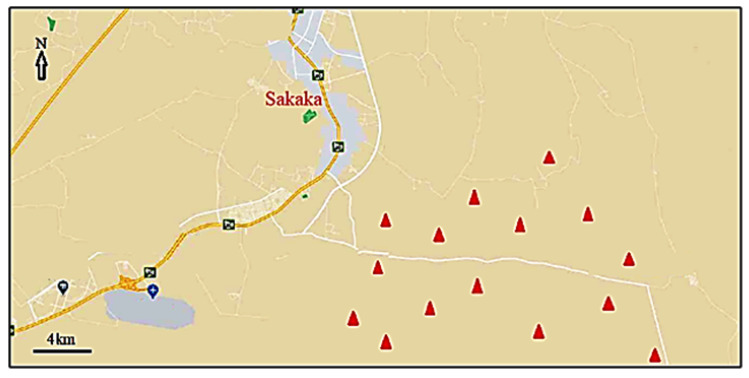
Map of the study area showing the sampled stands (triangles).

The study region is a sandy desert, representing the northern reaches of the Nafud desert, the second largest (after the Rub Al Khali) sand-dune desert in Saudi Arabia. The study area (Figure 5) is located 10–30 km south east of Sakaka city (29°58′11.06″ N 40°12′23.08″ E) in Al-Jouf Region, northwestern Saudi Arabia. The climate is hyper-arid with 55 mm mean annual rainfall (average from 2000–2010). The amount of yearly rainfall is variable. The rainy period and, accordingly, the growing season, extends mainly from November to May. The region is characterized by hot summers and cool winters. The mean monthly air temperature varied between 9.8 °C occurring in January and 33.8 °C in August. The lowest and highest average monthly relative humidity are 16% in June and 53% in January, respectively (Al-Jouf meteorological station).

### 4.2. Haloxylon salicornicum

*H. salicornicum* (Chenopodiaceae) is a diffuse, rounded, many-branched shrub, 60–100 cm high. Branches are thick and jointed and fleshy when young. Leaves are largely absent, reduced to minute scales. The distribution range covers Northern Africa and Asia in sandy and stony deserts [53,54]. It is widely distributed in Egypt, Saudi Arabia, Palestine, Jordan, Iraq, Iran, Pakistan, India and Kuwait [55]. The species is native in Saudi Arabia where it occurs in a variety of habitats, including wadi-terraces, sandy plains, and gravel deserts [56]. It is adapted to survive under severe environmental stresses including drought, salinity and overgrazing in arid regions [57]. *Rimth* saltbush shrubland, dominated by *H. salicornicum,* likely covers more area than any other single plant community in northeastern Arabia [58]. The plant could be utilized for vegetation restoration and fixation of sand dunes [36]. It is also used as firewood, as a source of bioactive phytochemicals with pharmaceutical significance and food source for domestic stock and wildlife [55].

### 4.3. Shrub Effects on Annual Species

A total of 15 stands (40 m × 40 m each) were established at the study area. Stands were selected to cover variations in the annual vegetation within the study area. At each stand, 10 *H. salicornicum* shrubs and 10 open areas nearby were selected randomly. A quadrat (1 m × 1 m) was located randomly below each selected shrub and a similar quadrat was laid randomly in the open area adjacent to the selected shrub, giving a total of 20 permanent quadrats per stand. The open area was at least 2 m away from the canopy edge of any shrub. Individuals of each annual species present in the quadrats were counted in March of the four growing seasons, 2015–2016, 2016–2017, 2017–2018 and 2018–2019. For each microhabitat (under shrub and open area) per stand per growing season, the density of individual species was determined as the number of individuals of a given species/m^2^ and the total density of plants was estimated as the number of individuals of all species/m^2^, while the species richness was measured as the total number of species present.

### 4.4. Soil Analysis

In March of each growing season, three soil samples (0–30 cm depth) were collected randomly from each microhabitat per stand. The three samples per microhabitat per stand per growing season were pooled together forming one composite sample. For soil texture analysis, a measured 100 g of soil was passed through a 0.05 mm sieve to separate sand (>0.05 mm) and silt + clay (<0.05 mm). Electrical conductivity and pH of 1:5 (*w*/*v*) soil-water extract were determined using electrical conductivity meter, and pH meter, respectively. Oxidizable soil organic carbon content was measured using the Walkley and Black procedure. Soil moisture content was estimated by drying soil in an oven at 105 °C for 48 h. Available nitrogen was determined by the micro-Kjeldahl method, while available phosphorus was estimated via the Olsen method, using sodium bicarbonate as an extracting agent. The available potassium content was measured using a flame photometer. Soil analyses were performed according to procedures outlined by Black [59] and Gupta [60].

### 4.5. Data Analysis

A general linear model (GLM) was used to test the effects of shrub (under shrub and open area) and growing season on total density and species richness of annual plants. Relative interaction indices (RII) [61] were used to assess the effects of *H. salicornicum* shrubs on the community attributes, the total density of plants and species richness. RII = (CA_u_ − CA_o_)/(CA_u_ + CA_o_), where CA_u_ and CA_o_ are the community attribute under shrub and in open area next to it, respectively. RII values range from −1 to +1. Negative values indicate negative effects of shrubs on annual plants, positive values show facilitative effects, and a 0 value displays a neutral effect. The one-sample *t*-test was used to check whether RII values differ significantly from zero. The independent samples *t*-test was used to compare between the values of total plant density, species richness and soil properties of open area versus under shrubs.

Ordination of observations (the sampled stands in all growing seasons) by Detrended Correspondence Analysis (DCA) [62] based on the density of individual species was used to estimate the effects of *H. salicornicum* shrubs and growing season on the structure of annual plants communities. Observations lying close to each other in the ordination plot were considered similar in terms of their community structure, and vice-versa. To test whether the scattering of observations in the ordination plot was significantly influenced by shrub and growing season, a general linear model was applied to assess the effects of shrub and growing season on the scores of the first two DCA axes [63]. The general linear model was also used to estimate the influence of shrub and growing season on soil properties. The general linear model and *t*-test were performed using SPSS v.16 software (SPSS, Chicago, IL, USA).

## 5. Conclusions

The present study indicates that the facilitative effects of *H. salicornicum* shrubs on their sub-canopy annual plants shift to negative under extreme drought conditions. These results did not support the stress-gradient hypothesis but do agree with its recent modifications. The divergence of our results from predictions of the stress-gradient hypothesis could be related to the reversed changes in the effects of shrubs on soil moisture with growing-season rainfall. Shrubs exerted negative effects on sub-canopy wetting in dry growing seasons, but improved understory soil moisture content in the wet growing season. Our study assumes that facilitation by shrubs may shift to negative effects with the increase in aridity predicted in the coming decades. Therefore, increased drought due to climate change may have negative consequences on plant diversity in arid regions.

## Figures and Tables

**Table 1:** General linear model analysis for the effects of shrub (under shrub vs. open area) and growing season on total density and species richness of annual plants. F-values are shown.

Factor	Total Density	Species Richness
Shrub	20.750 ***	26.970 ***
Growing season	1033.564 ***	745.043 ***
Shrub × growing season	138.365 ***	109.877 ***

***, *p* < 0.001.

**Table 2:** Mean relative interaction index (RII) values ± SD as indication for the effects of *Haloxylon salicornicum* shrubs on the total density and species richness of annual plants in four growing seasons. *, RII values differ significantly (*p* < 0.05) from zero according to one-sample *t*-test.

Growing Season	Total Density	Species Richness
2015–2016	−0.27 ± 0.17 *	−0.26 ± 0.09 *
2016–2017	−0.31 ± 0.05 *	−0.27 ± 0.08 *
2017–2018	−0.31 ± 0.03 *	−0.30 ± 0.10 *
2018–2019	0.26 ± 0.03 *	0.16 ± 0.04 *

**Table 3:** Effects of *Haloxylon salicornicum* shrubs on the density of annual species in four growing seasons as indicated by relative interaction index (RII) values ± SD. *, RII values differ significantly (*p* < 0.05) from zero according to one-sample *t*-test.

Species	2015–2016	2016–2017	2017–2018	2018–2019
*Agriophyllum minus* Fisch. and C.A.Mey.	−0.80 ± 0.28 *	−0.93 ± 0.16 *	−0.75 ± 0.50 ns	0.53 ± 0.26 *
*Aizoon hispanicum* L.				–
*Anastatica hierochuntica* L.	–			0.11 ± 1.02 ns
*Anisosciadium lanatum* Boiss.				–
*Anthemis haussknechtii* Boiss. and Reut.	−0.48 ± 0.26 *	−0.75 ± 0.24 *	−0.46 ± 0.30 *	0.28 ± 0.11 *
*Arnebia decumbens* (Vent.) Coss. and Kralik		–		0.67 ± 0.58 ns
*Asteriscus hierochunticus* (Michon) Wiklund				0.67 ± 0.47 ns
*Astragalus annularis* Forssk.	−0.83 ± 0.29 *			0.16 ± 0.58 ns
*Bassia eriophora* (Schrad.) Asch.	−0.75 ± 0.35 ns			0.67 ± 0.47 *
*Bassia muricata* (L.) Asch.	−0.62 ± 0.30 *	−0.67 ± 0.58 ns	−0.83 ± 0.41 *	0.28 ± 0.13 *
*Brassica tournefortii* Gouan	–			0.44 ± 0.51 ns
*Cakile arabica* Velen. and Bornm.				–
*Calendula tripterocarpa* Rupr.		–		–
*Cleome amblyocarpa* Barratte and Murb.				0.67 ± 0.58 ns
*Cutandia memphitica* (Spreng.) K.Richt.				–
*Diplotaxis acris* (Forssk.) Boiss.	–	−0.67 ± 0.58 ns	–	0.45 ± 0.41 *
*Erodium laciniatum* (Cav.) Willd.	−0.56 ± 0.38 ns	−0.50 ± 0.71 ns	−0.95 ± 0.15 *	0.40 ± 0.34 *
*Horwoodia dicksoniae* Turrill			−0.75 ± 0.35 ns	0.84 ± 0.36 *
*Ifloga spicata* (Forssk.) Sch.Bip.	−0.75 ± 0.28 *	−0.75 ± 0.35 ns	−0.75 ± 0.50 ns	0.26 ± 0.08 *
*Limonium lobatum* (L.f.) Chaz.				0.67 ± 0.47 ns
*Malva parviflora* L.	−0.83 ± 0.41 *	−0.75 ± 0.50 ns	−0.50 ± 0.71 ns	0.43 ± 0.38 *
*Matthiola longipetala* (Vent.) DC.				0.42 ± 0.69 ns
*Medicago laciniata* (L.) Mill.				0.50 ± 0.71 ns
*Mesembryanthemum nodiflorum* L.	–			0.33 ± 1.15 ns
*Neurada procumbens* L.			–	0.50 ± 0.71 ns
*Opophytum forsskalii* (Hochst. ex Boiss.) N.E.Br.				0.50 ± 0.64 ns
*Paronychia arabica* (L.) DC.		−0.50 ± 0.71 ns		0.75 ± 0.50 ns
*Plantago boissieri* Hausskn. and Bornm.	−0.14 ± 0.04 *	−0.14 ± 0.03 *	−0.14 ± 0.04 *	0.22 ± 0.08 *
*Plantago amplexicaulis* Cav.	–	–	–	0.75 ± 0.31 *
*Plantago ciliata* Desf.	−0.95 ± 0.11 *	−0.67 ± 0.47 ns	−0.67 ± 0.58 ns	0.29 ± 0.62 ns
*Plantago ovata* Forssk.	−0.92 ± 0.18 *	−0.67 ± 0.58 ns	−0.80 ± 0.45 *	0.59 ± 0.40 *
*Pteranthus dichotomus* Forssk.				–
*Rumex vesicarius* L.	−0.63 ± 0.48 *	−0.67 ± 0.58 ns	–	0.63 ± 0.41 *
*Savignya parviflora* (Delile) Webb	–		–	0.61 ± 0.31 *
*Schimpera arabica* Hochst. and Steud. ex Steud.				0.50 ± 0.71 ns
*Schismus barbatus* (L.) Thell.	−0.27 ± 0.13 *	−0.54 ± 0.12 *	−0.52 ± 0.15 *	0.19 ± 0.05 *
*Spergularia bocconei* (Scheele) Graebn.	–			0.38 ± 0.95 ns
*Trigonella stellata* Forssk.	−0.5 ± 0.71 ns	–		0.72 ± 0.36 *

Empty cells mean that species not present; –, species excluded from analysis as they occurred in less than 10% of stands in a growing season. ns, non-significant.

**Table 4:** General linear model analysis for the effects of shrub (under shrub vs. open area) and growing season on the position of observations in the DCA plot using the scores of the first two DCA axes. F-values are shown.

Factor	DCA Axis 1	DCA Axis 2
Shrub	90.141 ***	0.365 ns
Growing season	250.725 ***	0.800 ns
Shrub × growing season	15.395 ***	0.078 ns

***, *p* < 0.001; ns, non-significant.

**Table 5:** General linear model testing the effects of shrub (under shrub vs. open area) and growing season on soil properties. F-values are shown.

Parameter	Shrub	Growing Season	Shrub × Growing Season
Organic carbon (%)	36.257 ***	0.097 ns	0.031 ns
Moisture content (%)	14.215 ***	1594.726 ***	21.923 ***
Electrical conductivity (dS/m)	457.219 ***	1.795 ns	0.015 ns
pH	1.812 ns	0.395 ns	0.663 ns
Sand	1378.504 ***	0.020 ns	0.043 ns
Silt + clay	1378.504 ***	0.020 ns	0.043 ns
N (mg/kg)	75.045 ***	0.034 ns	0.102 ns
P (mg/kg)	1932.392 ***	0.025 ns	0.190 ns
K (mg/kg)	681.917 ***	0.021 ns	0.039 ns

***, *p* < 0.001; ns, non-significant.

**Table 6:** Soil characters under shrubs of *Haloxylon salicornicum* and in open area. Values are means ± SD. Microhabitats in a given growing season sharing the same letter are not significantly different at *p* < 0.05 according to independent samples *t*-test.

Soil Variable	2015–2016		2016–2017		2017–2018		2018–2019	
	Under Shrub	Open Area	Under Shrub	Open Area	Under Shrub	Open Area	Under Shrub	Open Area
Organic carbon (%)	0.34 ± 0.09 a	0.26 ± 0.08 b	0.33 ± 0.08 a	0.25 ± 0.08 b	0.35 ± 0.08 a	0.25 ± 0.08 b	0.34 ± 0.08 a	0.24 ± 0.08 b
Moisture content (%)	1.32 ± 0.18 b	1.53 ± 0.21 a	1.18 ± 0.16 b	1.35 ± 0.18 a	1.19 ± 0.15 b	1.37 ± 0.19 a	4.77 ± 0.30 a	4.22 ± 0.31 b
Electrical conductivity (dS/m)	0.76 ± 0.04 a	0.57 ± 0.06 b	0.78 ± 0.04 a	0.58 ± 0.05 b	0.77 ± 0.04 a	0.58 ± 0.06 b	0.75 ± 0.04 a	0.56 ± 0.06 b
pH	7.87 ± 0.14 a	7.85 ± 0.12 a	7.93 ± 0.15 a	7.86 ± 0.08 a	7.87 ± 0.12 a	7.85 ± 0.12 a	7.90 ± 0.11 a	7.87 ± 0.11 a
Sand (%)	86.3 ± 0.99 b	92.2 ± 0.79 a	86.1 ± 1.03 b	92.3 ± 0.72 a	86.2 ± 1.04 b	92.4 ± 0.73 a	86.1 ± 1.04 b	92.3 ± 0.74 a
Silt + clay (%)	13.7 ± 0.99 a	7.8 ± 0.79 b	13.9 ± 1.03 a	7.7 ± 0.72 b	13.8 ± 1.04 a	7.6 ± 0.73 b	13.9 ± 1.04 a	7.7 ± 0.74 b
N (mg/kg)	108.6 ± 8.0 a	97.1 ± 7.8 b	108.7 ± 7.8 a	97.4 ± 8.0 b	109.1 ± 7.9 a	95.8 ± 7.5 b	109.0 ± 7.1 a	96.8 ± 6.9 b
P (mg/kg)	2.81 ± 0.12 a	1.65 ± 0.16 b	2.80 ± 0.11 a	1.64 ± 0.16 b	2.79 ± 0.12 a	1.65 ± 0.16 b	2.78 ± 0.12 a	1.68 ± 0.17 b
K (mg/kg)	123.7 ± 6.8 a	85.5 ± 9.4 b	124.0 ± 6.6 a	86.0 ± 8.9 b	123.6 ± 7.1 a	85.4 ± 9.4 b	124.3 ± 6.2 a	84.9 ± 9.2 b
